# Multi-Contextual Segregation and Environmental Justice Research: Toward Fine-Scale Spatiotemporal Approaches

**DOI:** 10.3390/ijerph14101205

**Published:** 2017-10-10

**Authors:** Yoo Min Park, Mei-Po Kwan

**Affiliations:** Department of Geography and Geographic Information Science, University of Illinois at Urbana-Champaign, Natural History Building, MC-150, 1301 West Green Street, Urbana, IL 61801, USA; mkwan@illinois.edu

**Keywords:** multi-contextual segregation, environmental justice, uncertain geographic context problem, fine-scale spatiotemporal approaches, Big Data, environmental exposure, environmental health hazards, health disparities, human mobility, time geography

## Abstract

Many environmental justice studies have sought to examine the effect of residential segregation on unequal exposure to environmental factors among different social groups, but little is known about how segregation in non-residential contexts affects such disparity. Based on a review of the relevant literature, this paper discusses the limitations of traditional residence-based approaches in examining the association between socioeconomic or racial/ethnic segregation and unequal environmental exposure in environmental justice research. It emphasizes that future research needs to go beyond residential segregation by considering the full spectrum of segregation experienced by people in various geographic and temporal contexts of everyday life. Along with this comprehensive understanding of segregation, the paper also highlights the importance of assessing environmental exposure at a high spatiotemporal resolution in environmental justice research. The successful integration of a comprehensive concept of segregation, high-resolution data and fine-grained spatiotemporal approaches to assessing segregation and environmental exposure would provide more nuanced and robust findings on the associations between segregation and disparities in environmental exposure and their health impacts. Moreover, it would also contribute to significantly expanding the scope of environmental justice research.

## 1. Introduction

Human exposure to environmental harm is a function of various socioeconomic processes that push people toward the threshold of environmental stressors, rather than just a product of accidental environmental impacts [1]. Numerous studies have reported that racial and ethnic minorities or socioeconomically disadvantaged people are exposed to greater environmental harm [2,3,4,5,6,7,8,9,10]. This phenomenon has been well documented in environmental justice literature and has been variously termed environmental injustice, environmental racism, or environmental inequality [9]. This paper uses the term environmental justice to refer to the “equal access to a clean environment and equal protection from possible environmental harm irrespective of race, income, class, or any other differentiating feature of socio-economic status” [11] (p. 13).

A number of quantitative environmental justice studies have sought to understand the association between residential segregation and disparities in exposure to environmental health hazards (e.g., air pollution) or health benefits (e.g., green space) among different social groups. However, the heavy use of aggregate data and the narrow focus solely on residential contexts in past studies have led to several methodological problems as well as inconsistent findings on the association. In this review paper, we discuss the limitations of residence- and place-based approaches in segregation and environmental exposure assessment. We then suggest that future environmental justice research should consider spatiotemporal population dynamics and regard individuals as mobile agents in urban spaces in order to address the complexity of the dynamic sociospatial mechanisms underlying unjust environmental exposure among social groups. This in turn calls for a reconceptualization of and new measures for segregation, as well as a redelineation of the individual geographic and temporal contexts in which people actually experience segregation and unequal exposure to environmental factors.

## 2. Residential Segregation and Disparities in Environmental Exposure

Early environmental justice scholars focused largely on the residential neighborhoods of socially marginalized people and their proximity to noxious resources and facilities [2,12,13,14,15], yet they were engaged in a “chicken-or-egg” debate—whether marginalized people move to an area before toxic chemical sources are introduced, or their communities were intentionally established in areas where toxics already existed [13,16,17,18,19,20]. Helfand and Peyton [14] argued that toxic facilities would likely be established in disadvantaged areas due to their low property and land values and the increased likelihood that socioeconomically marginalized residents in these areas would accept the proximity of such facilities with less compensation. Bullard [2,12,13] found that many hazardous facilities, including landfills, toxic chemical sites and lead smelters, were disproportionately constructed in residential neighborhoods with high percentages of African Americans, despite the smaller proportion of African Americans relative to other racial groups living in the city at large. However, Bullard [2] also noted that socially marginalized groups tend to “choose” neighborhoods where hazardous facilities already exist, due in part to low housing costs. Accordingly, he found that their housing choices tended to be based on cost rather than environmental quality. The polarization of the early debate compelled environmental justice researchers to focus on residential locations and their spatial proximity to toxic facilities.

As an extension of these residence-based studies in the vast environmental justice literature, several researchers argued that residential segregation may be one of the fundamental factors perpetuating inequalities in environmental exposure and health [21,22,23,24,25,26,27,28,29]. Residential segregation refers to the geographic separation of a specific social group from another in a residential context [30]. This practice results from systemic, structural and complex discriminatory processes, such as discriminatory housing practices supported by the federal government, uneven distribution of educational and employment opportunities between inner cities and suburbs, the ideology of white supremacy and uneven industrial development, all of which affect people’s well-being [24,25,29,31,32,33,34].

There has been a long history of measuring residential segregation by developing indices in sociology and demography [35], but it was only in the early 21st century that researchers began to use measures of residential segregation for environmental justice or environmental health issues [22,23,24,25,26,28,29,32,36,37,38]. William and Collins [29] viewed residential segregation as a fundamental cause of health disparities among racial groups insofar as it adversely impacts access to education and job opportunities as well as social and physical environments. Extending this argument, Schulz et al. [28] suggested a conceptual framework in which residential segregation is a primary determinant of the risk of cardiovascular disease. The authors argued that social and physical environments serve as intermediate factors that modify relationships between residential segregation and factors more directly associated with health outcomes, such as health behaviors, exposure to environmental and social stressors and psychological factors. Similarly, Landrine and Corral [32] presented three ways in which residential segregation may lead to health disparities among African Americans: higher exposure to air pollution, lower healthcare quality and poorly constructed residential neighborhoods. The authors concluded that to better understand racial disparities in environmental health, researchers need to focus on the characteristics of local contexts/places in which racial groups reside, rather than on just their racial characteristics or cultures.

Although the literature has increasingly linked residential segregation to social disparities in environmental exposure and associated health risks, recent studies have noted that it is still unclear whether segregation is significantly associated with such disparities [36,39]. Research findings on this relationship have thus far been inconsistent [39]. Using air pollution as an example of an environmental health risk factor, the next section reviews previous studies that have yielded different results on the association between segregation and disparities in environmental health risk.

## 3. Discrepancies in Research Findings in the Literature

Previous empirical studies on the association between residential segregation and unequal exposure to air pollution have reported inconsistent results, ranging from a strong association to no association. Using the dissimilarity index––the most commonly used measure of residential segregation––Lopez [23] found that African Americans tended to live in census tracts with higher levels of total ambient air toxics than whites in every large metropolitan area in the U.S. The author suggested that the observed strong association between residential segregation and unequal exposure to air toxics may be due to disproportionately located pollution sources as a consequence of racism and African Americans’ limited mobility entrapping them in polluted residential areas. In another study using the same segregation index, the relationship between average metropolitan air pollution levels and values of the segregation index in metropolitan statistical areas (MSAs) differed by pollutant [24]. The authors observed that segregation of whites from African Americans was positively associated with sulfur dioxide and ozone but negatively associated with carbon monoxide and nitrogen oxides; no significant association was found with particulate matter less than 10 micrometers in diameter (PM_10_). These mixed associations, however, may be due in part to the size of the spatial unit used to derive relevant variables (i.e., MSAs). An MSA may be too large to capture small-scale spatial variations with respect to some air pollutants, and the resulting imprecise macro-scale analysis may lead to erroneous results [40].

Unlike the aforementioned studies, which focused only on a dyadic racial comparison, Morello-Frosch and Jesdale [37] utilized the multi-group dissimilarity index to examine the association between residential segregation and health risks associated with exposure to air pollution. The authors found that cancer risk associated with hazardous air pollutants grew with increasing levels of residential segregation for all racial and ethnic groups, but the relationship was strongest for Hispanics. A more recent study found that air pollution-related cancer risk increased in census tracts with higher proportions of African Americans [22]. However, as many studies have pointed out that percentage is not an appropriate segregation measure [41,42,43], the use of percentages of African Americans in the study raises the question of whether high percentages of a certain group necessarily reflect a high level of segregation. A higher percentage of a particular racial group could merely indicate the areal dominance of that group or diversity across the area if any other racial groups are also highly concentrated in the same area [43]. Oka and Wong [44] argued that simply using racial proportions as a measure of segregation makes it harder to understand pathways through which segregation affects health outcomes.

Contrary to the findings of these studies, however, several empirical studies concluded that the association between residential segregation and environmental health disparities is either unclear or nonexistent. For instance, Downey [45] found that highly segregated cities in the U.S. metropolitan areas did not correspond to cities with the highest racial disparities in terms of exposure to air pollution. The author argued that minorities are not always concentrated in areas with high air-pollution levels, and that polluting sources may be evenly distributed across urban areas. As an extension of this study, Downey et al. [46] used the dissimilarity index and average toxic levels for metropolitan areas in the U.S. to demonstrate that metro-level residential segregation did not adequately explain disparities in environmental exposure. A more recent study using the same index also observed no association between racial residential segregation and disparities in lifetime cancer risk associated with air pollution in Charleston, South Carolina [38]. Rather, the authors found that economic deprivation was more closely associated with all sources of lifetime cancer risk than racial residential segregation. This finding contradicts Lopez’s [23] conclusion that income disparity is a relatively poorer predictor of inequality in environmental exposure than racial disparity. Clark et al. [47] also arrived at the conclusion that racial disparity had a twofold greater association with unequal exposure to nitrogen dioxide (NO_2_) than income disparity.

This paper argues that the mixed findings regarding the association between segregation and environmental exposure are due to several methodological problems usually found in residence- and place-based approaches in environmental justice research. The next section discusses these methodological problems and how they can lead to misleading research findings.

## 4. Methodological Problems in Segregation and Environmental Justice Research

### 4.1. Limited Incorporation of Geographic Principles in Assessing Segregation and Environmental Exposure

For decades, segregation studies have developed various indices to measure levels of residential segregation, with most early works completed by sociologists and demographers [35]. It was only after the 1990s that geographers assumed a more explicit role in the segregation literature by integrating the spatial dimension into measures of segregation. Due to the limited involvement of geographers in the history of segregation studies, the vast majority of the literature has separated social processes from spatial processes, even though segregation is a complex sociospatial phenomenon, with spatial aspects receiving less scholarly attention. Many traditional measures have tried to quantify the five dimensions of segregation defined by Massey and Denton [41]—evenness, exposure (or isolation), centralization, concentration and clustering (The five dimensions of segregation are defined as follows (for more detailed explanations, see Massey and Denton [41]): (1) evenness: the differential distribution of social groups; (2) exposure (or its counterpart, isolation): the possibility of interaction between social groups; (3) centralization: the extent to which a group is located near the city center; (4) concentration: the share of urban space occupied by a group (density of a group in urban space); and (5) clustering: the degree of spatial proximity of social groups.)—but some recent studies have pointed out that these indices do not adequately address the spatial patterns of residence of different population groups [35,39,42,43,44,48,49,50,51,52,53].

For example, the dissimilarity index (*D*), developed by Duncan and Duncan [54], measures the evenness dimension of segregation and can be interpreted as the proportion of a minority group that would need to move to another area (e.g., census tract) to achieve an even distribution of that group throughout an entire metropolitan area (The dissimilarity index (*D*) is calculated by the following equation [54]: $D=\frac{1}{2}\sum \left|\frac{A_{i}}{A}-\frac{B_{i}}{B}\right|$, where A is the total population of group A in an entire city; $A_{i}$ is the population of group A in *i*th sub-areal unit; B is the total population of group B in the entire city; and $B_{i}$ is the population of group B in *i*th sub-areal unit). Although this index has been the most commonly used, even until recently due to its easy calculation [35,43], it has long been criticized as an “aspatial” measure that only considers the racial/socioeconomic composition within each areal unit and is thus incapable of capturing spatial relationships between areal units or population groups [43,48].

**Figure 1:**
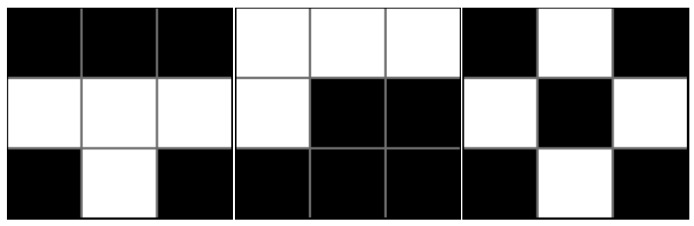
The checkerboard problem in traditional measures: All the cases above have the same value in a segregation index (e.g., *D* = 1) despite the different spatial arrangements of spatial units.

White [55] articulated this aspatial attribute by suggesting two methodological problems of traditional indices: (1) the checkerboard landscape problem, and (2) the modifiable areal unit problem (MAUP) [56]. With regard to the checkerboard problem, imagine a checkerboard landscape on which two population groups live exclusively on alternating squares. In aspatial measures, even though each of the squares (e.g., a census tract) is rearranged with any spatial patterns within the entire landscape (e.g., a metropolitan area), the value of the segregation index will not change if each square is still occupied exclusively by one of the two population groups [55]. The value still indicates perfect segregation (e.g., *D* = 1) because it is affected only by the population mix within each spatial unit (e.g., census tract), not by spatial patterns between the units across the entire region (Figure 1). This thus raises the question of whether traditional indices can accurately measure the actual level of segregation.

The MAUP, which was proposed by Openshaw [56], is a well-known methodological problem in geography and spatial analysis. The MAUP arises when artificially delineated areal units are used to analyze geographically continuous phenomena. The value of a segregation index may be inconsistent depending on which areal unit is used to calculate it because the values are affected by the size of the areal unit (i.e., the scale effect)—such as block groups versus census tracts—and the way of groupings at a given scale (i.e., the zoning effect)—such as health professional shortage areas versus health service areas. Many studies on how to measure segregation have relied on area-level data because population data are usually collected based on administrative units, such as census tracts, and because such data are easy to use and interpret [42,57]. Wong [50] found that when evaluated based on smaller spatial units, the level of segregation tended to be higher than when larger units were used.

These problems apply to all traditional indices that use population counts that are aggregated within arbitrarily delineated spatial units and that do not consider spatial patterning (or spatial contiguity) of the units. Due to such limitations in traditional measures of residential segregation, some scholars have cast doubt on using indices to understand segregation. Johnston et al. [58] pointed out that all traditional global measures are no more than single numbers. Such summary measures do not yield a comprehensive understanding of the geographic configuration of a socioeconomic/ethnic residential mosaic because a significant number of important spatial details are lost when the index value is calculated. Also, although the dissimilarity index, which is used to measure the evenness dimension of segregation, has been widely incorporated in many studies on the relationship between segregation and environmental health risk [39,59], why this dimension was considered the most appropriate for environmental health research has rarely been discussed [39].

Some efforts have been made to develop spatial segregation measures (e.g., [42,60,61,62,63]). Johnston et al. [58] suggested using geographical approaches to better reflect the spatial nature of segregation, such as measures of local spatial autocorrelation, instead of using single-number global measures. In a study that examined the association between exposure to ambient air pollution and racial residential segregation, Jones et al. [36] utilized the Getis and Ord Gi* statistic [64,65] to identify the level of spatial clustering in U.S. census tracts in which a particular racial/ethnic group was concentrated (The Gi* statistic returns a z-score, which indicates which areas with low or high attribute values cluster spatially. If an area has a high z-score, it means that its neighboring areas also have high attribute values, and vice versa. The statistic is calculated by the following equation: $G_{i}^{*}=\frac{\sum_{j=1}^{n}w_{ij}x_{j}}{\sum_{j=1}^{n}x_{j}}$, where $w_{ij}$ is the spatial weight value between areas *i* and *j* (which indicates their spatial relationship); $x_{j}$ is the attribute value of area *j*; and *n* is the total number of areas.). Similarly, a more recent study used the same spatial statistical method to investigate the joint effects of racial segregation and air pollution on cardiovascular outcomes [66]. However, some scholars have been skeptical about whether spatial autocorrelation and local spatial statistical approaches can improve the measurement of segregation levels [35,67], arguing that a high degree of positive spatial autocorrelation does not always indicate a high level of segregation. For example, even if a particular social group is highly concentrated in some nearby areas and thus has a high level of local spatial autocorrelation, we cannot say that this group is highly segregated if any other social group is also clustered in the same areas.

Despite many efforts to “spatialize” segregation measures, it remains unclear whether such measures produce more reliable and accurate results than aspatial measures in segregation-related research and whether they involve a conceptually and theoretically agreeable meaning of segregation [35,42]. Although spatial measures account for the spatial dimension of segregation to some degree, we argue that they also have a major shortcoming. Nearly all such measures define segregation as a phenomenon observed only in a residential context. In other words, these measures only take residential segregation into account, which is just one of numerous types of segregation that occur in the multiple geographic contexts of people’s everyday lives, including the workplace and social/recreational venues. For this reason, segregation measures, be they spatial or aspatial, that ignore the multi-dimensional aspects of segregation may lead to a biased understanding of people’s segregation experiences [68,69].

Meanwhile, many previous environmental justice studies on the effect of segregation have relied on spatially and temporally aggregated environmental data. However, if spatiotemporally continuous environmental risk factors, such as air pollution, are artificially aggregated into areal units, the MAUP can also result. For example, Rice et al. [38] used census tract-level data for lifetime cancer risk associated with air toxics, which were estimated from the national-scale air toxics assessment (NATA), in order to examine their association with census tract-level segregation index values. Many other studies have also used NATA data (e.g., [4,18,22,37,70,71,72,73]). Aggregate-level air pollution exposure data have also been employed in other studies (e.g., [23,24,46]).

Such a limited incorporation of geographic principles in the assessment of the two spatial phenomena—segregation and environmental exposure—can cause several methodological problems in environmental justice research. However, we argue that the most important but least recognized methodological problem in the literature is that the research has focused only on the residential context, even though it may not represent the true geographic context in which individuals experience segregation and disproportionate environmental impacts. Therefore, a significant amount of uncertainty remains in research findings because they do not tell us about people’s experience of segregation and environmental exposure outside of their residential areas [69,74]. We discuss this problem in more detail in the next subsection.

### 4.2. The Uncertain Geographic Context Problem (UGCoP)

While both aspatial and spatial measures of residential segregation have contributed to quantifying the degree of residential separation among different social groups, segregation experienced in various other daily activity locations has been less examined. In previous studies, the basic assumption underlying the use of residential segregation measures was that people are non-mobile and face segregation only in residential contexts; accordingly, people living in the same residential areas would be expected to experience the same levels of segregation over the course of a day. As a result, attempts to determine the association between segregation and environmental justice issues have also been bound to residential contexts. Yet, focusing only on residential neighborhoods can produce a considerable amount of uncertainty in research results, given that people spend a significant amount of time outside their homes. Kwan [75] defined this issue as the uncertain geographic context problem (UGCoP).

The UGCoP refers to the problem that research results about the association between contextual factors and people’s health and behaviors may be erroneous when individuals’ geographic and temporal contexts are misrepresented yet nonetheless used to derive the relevant contextual variables [75,76]. This problem arises when conventional areal units (e.g., census tracts) do not correspond to people’s true geographic contexts [75]. Failing to consider the UGCoP could lead to serious inferential errors or misleading findings, such as false negative or false positive associations [75,77]. Nevertheless, most quantitative studies on the relationship between segregation and air pollution-related health risks have paid very little attention to the UGCoP. In this paper, we argue that the resultant erroneous findings have generated uncertain conclusions about the association between segregation and social disparities in environmental exposure and health risk in the literature.

That said, some more recent studies have recognized the UGCoP as an important methodological issue and the need to mitigate its effects on research findings (e.g., [40,78,79,80,81,82]). Approximating an individual’s true spatiotemporal context is especially crucial in studies on exposure to air pollution due to the highly dynamic characteristics of both air pollution and human beings [40,74,83,84,85,86,87,88,89,90,91,92]. Since air pollution concentrations constantly change and humans are mobile across both space and time, various levels of air pollution may be experienced at different moments as well as in residential neighborhoods, workplaces and recreational venues [40,75,76]. Without considering such dynamism, research findings may be corrupted by the UGCoP.

One way to mitigate the UGCoP is to use mobile tracking technology, such as global positioning systems (GPS), to identify people’s true geographic and temporal contexts [75]. GPS can collect precise, high-resolution information about people’s movements in space and time, enabling us to know not just their exact residential locations but also where and when they work, shop and do leisure activities. The spatiotemporal contexts in which individuals move or perform daily activities better correspond to the true contexts in which they are affected by environmental or contextual factors [75,77,93,94,95]. GPS data can be used in tandem with survey data about individuals’ activities and travels, such as destinations, start/end times, activity types (trip purposes) and transportation modes. Note that these methods can considerably mitigate the effects of the UGCoP as well as the other methodological problems (i.e., the checkerboard problem and the MAUP) [42]. This means that by addressing all three major methodological problems, such fine-scale human movement data permit far more robust results. The value of detailed, individual-level data is further enhanced when combined with advanced geographic information science (GIS) methods [76]. Recent advances in three-dimensional (3D) GIS have also enabled researchers to better analyze and visualize large and complex spatiotemporal data like those collected from GPS or other location-aware devices.

Some recent environmental exposure studies have made use of the Big Data (which is also called “fine-scale spatial-temporal data” in spatial information sciences [96]) revolution over the last decade. For instance, Park and Kwan [40] used simulated individual-level movement data and found that individuals’ actual levels of exposure to air pollution can be either underestimated or overestimated if their daily mobility is not considered. Similar conclusions were also drawn in Setton et al.’s [97] study, which was based on transportation survey data; Yoo et al.’s [91] study, which used both activity-travel diary data and GPS data; de Nazelle et al.’s [98] study, which used smartphone-based movement and physical activity tracking data; and Su et al.’s [99] study, which used smartphone-based real-time location tracking data obtained from public WiFi networks. Furthermore, Jerrett et al. [100] demonstrated the great potential of wearable air-pollution sensors together with mobile phone tracking capabilities. These studies show how using human movement data with high spatiotemporal resolutions can modify research questions and designs, as well as how such data can help generate more reliable and realistic findings, mitigating the UGCoP.

However, while richly detailed individual-level data have significantly benefited environmental exposure/health studies via the increased accuracy of exposure assessments, environmental justice scholars have not yet adequately taken advantage of such benefit. It may be because of the challenges in obtaining or using such high-resolution data, including the significant amount of cost and time of data collection, data confidentiality, computational complexity and the need to protect research participants’ privacy [40,76] (Table 1). However, these limitations are addressable. Several activity-travel survey datasets and GPS datasets collected in major U.S. metropolitan areas are available at the Transportation Secure Data Center in the National Renewable Energy Laboratory at no cost. Confidentiality for geocoded individual-level data can be ensured with geographic masking techniques for minimizing the risk of reidentification of individuals, and personal privacy can be protected by suitable human subject protocols [40,101,102]. Advanced GIS and geospatial technologies have enabled us to analyze, store, manage and visualize such large, complex geospatial datasets [76]. Moreover, strategic sampling methods that are based on suitable geographic and socioeconomic stratifications would help obtain an adequate number of participants from all population groups at various localities of the study area (e.g., oversampling underrepresented groups). Efforts to address the limitations of fine-scale spatiotemporal approaches that use high-resolution data have been actively ongoing. Future environmental justice research should also focus on addressing these challenges in order to enhance the benefits of such approaches.

## 5. Beyond Residence- and Place-Based Approaches

### 5.1. Segregation in Various Daily Life Contexts

Traditionally, socioeconomic or racial/ethnic segregation has been a static notion closely associated with residential areas [69]. This notion, however, aggregates diverse individuals’ daily life spaces into the same residential areas or neighborhoods (e.g., the same census tract or block group). With the emergence of a “mobilities” paradigm within social science over the last decade [103,104], geographers and social scientists have recently started to reassess segregation as a dynamic concept, noting that people can experience segregation beyond their residential areas or neighborhoods [68,69,105,106,107,108,109,110]. Given that mobility affects people’s exposure to both various spatial and temporal contexts and different groups of people [111,112], it influences their spatiotemporal segregation experiences accordingly [69]. Mobility is more than just the actual distance traveled or the sum of trips and travel time [113,114]. Rather, mobility is affected by several factors: the availability of transportation modes, socioeconomic and racial constraints, spatial and temporal constraints, individual preferences, spatial distributions of services and activities, urban policies and designs, and so forth [111]. In general, socially marginalized groups tend to have more restricted daily mobility than other groups [109,111,115], because they tend not to own private vehicles and their residential neighborhoods are deprived of adequate public transportation, which spatially entraps them in resource-poor and environmentally unfavorable areas and limits their pursuit of a higher quality of life [18,113,116,117,118].

Recent studies have suggested that the scope of segregation-related research should be extended to include workplaces, grocery stores, or churches to better capture the dynamic experiences of segregation in various daily-life contexts [107]. This extension is supported by the argument that people living in the same residential area would not necessarily experience the same level of segregation [79,119,120,121,122]. Some studies have reported that different racial/ethnic groups tend to work in different urban areas. A century ago, workplaces were spatially tightly linked to residences because limited transportation modes reduced the maximum spatial range for commuting [106]. The clustering of racial/ethnic groups occurred as a result of slanted job referrals or familiar networks created via the immigration process or of ethnic-serving businesses around residential neighborhoods [123]. In contemporary urban areas, however, the difference in geographic patterns between home and work locations has become more prominent as more people can commute to non-residential areas due to advances in transportation modes, as well as urban processes such as gentrification, urban sprawl and the decentralization of employment [106,124]. This transformation has caused individuals’ segregation experiences to become more dynamic. Workplace segregation can be examined using the dissimilarity index (e.g., [106]), but a more sophisticated, comprehensive method is needed due to the aforementioned methodological problems of the index.

In addition to workplace segregation, another important but less recognized segregation type is free-time segregation. Different racial/ethnic groups tend to visit, for example, different parks [125], groceries [106] and churches [107,126], but activities based on common interests, such as sports, tend to attract people of diverse racial/ethnic backgrounds [127,128]. While some scholars have argued that people are less segregated in recreational places than at home or work because more spatial options for recreation exist [129], others have suggested that free-time segregation may occur through income, status identification, and social and ethnic networks [123]. For instance, low-income people tend to have fewer opportunities for leisure activities than high-income people [130], which may result in socioeconomic segregation during leisure time. Also, some people prefer to conduct free-time activities with their own social or ethnic group to preserve or strengthen their cultural identity and status [131,132]. Individuals’ workplace and residential social networks also influence where and with whom they spend leisure time because colleagues, neighbors, or co-ethnics are more likely to accompany them [123].

Along with this extended scope, the scale of segregation measures has also shifted from global measures (i.e., summary indicators for an entire study area [51,52]) to individual-level measures. The term “scale” in this context means the level of detail in the measure. Individual-level measures, if they are designed to use detailed human movement pattern data, can provide rich spatiotemporal details of individuals’ segregation experiences. Examining segregation at the individual level has shown great potential in some recent segregation studies (e.g., [35,68,79,110,133,134,135,136,137]). For instance, using an activity-travel survey dataset, Wong and Shaw [68] derived an activity space for each respondent by identifying a set of census tracts visited by that respondent, and then aggregated all the activity spaces based on his or her race/ethnicity. Then, the authors measured segregation levels using these aggregated activity spaces. Although this approach is an improvement over traditional segregation measures, it nonetheless continues to rely on residential population counts under the assumption that people are exposed to static residential populations in the census tracts they visit [57]. Silm and Ahas [137] addressed this limitation by using mobile phone data that included detailed daily movement patterns of Russians and Estonians in Estonia. Wang et al. [110] also extended Wong and Shaw’s [68] aggregated activity-space approach by decomposing activity space into three categories by activity type (i.e., working, shopping and recreation) using individual-level travel-behavior survey data in Beijing. Using 3D geovisualizations and analysis of variance, the authors showed that people from three socioeconomically different neighborhoods in Beijing had significantly different activity spaces in terms of extensity, intensity and exclusivity.

Considering individuals as active actors moving through the city, Netto et al. [134] visualized the daily movement trajectories of individuals and observed how different income groups in Niteroi, Brazil, have different mobility patterns and how they can be spatially co-present. In a study using social media data from Twitter and Foursquare, Shelton et al. [136] visualized the odds ratio between the number of tweets by East End people and West End people in Louisville, Kentucky to identify segregation during the daytime. The resulting map showed that nearly no tweets were posted from East End people in the West End, while tweets from both East and West End people in the East End were posted during the daytime. Temporal variations in segregation are also found in other recent studies (e.g., [79,137,138]). For example, Park and Kwan [79] developed an individual-level spatiotemporal proximity index to evaluate segregation at the individual level. Through geovisualizations of temporal variations in individuals’ segregation experiences in the greater Atlanta region, the authors showed that people experience different levels of segregation at different times of day. Such an index is especially useful because individuals’ segregation index values can be directly linked to various individual-level environmental variables, such as personal exposure to air pollution, green space and foodscape, or to health outcomes [79].

Despite the growing literature, however, there is still no consensus on the term used to describe this dynamic dimension of segregation in the literature [79]. Some studies have used separate terms for different activities, such as workplace segregation [106,123,139], occupational segregation [140,141], or free-time segregation [123]. In the meanwhile, the more comprehensive terms have been proposed, such as “time-space trajectories of segregation” [105] (p. 877), or “activity-space segregation” (e.g., [110,142,143]). The former term may be appropriate for studies using mobile tracking data with a time-geographic framework given that the word “trajectory” typically means the path of an object moving through continuous space-time from one destination to another. However, it may not be proper for studies utilizing spatiotemporally discrete data, such as activity-travel survey data, or using activity-space approaches (polygon-based approaches), such as standard deviational ellipses or minimum convex polygons. The latter term does not also embrace time-geographic approaches since the concept of activity-space—“the subset of all locations within which an individual has direct contact as a result of his or her day-to-day activities” [144] (p. 279)—does not necessarily include an explicit “time” component [68]. Therefore, the temporal context may be less emphasized in this term and thus temporal uncertainties may remain in research findings [79]. For example, Jones and Pebley [119] examined the spatial dimension of activity-space segregation (their term), but its temporal dimension was not considered. The same problem is also found in Schönfelder and Axhausen’s [118] study.

Noting the need for a more comprehensive term that can embrace both time-geographic and activity-space approaches, Park and Kwan [79] recently suggested a new notion of segregation, called multi-contextual segregation, in order to better describe the full spectrum of individual segregation experiences. Multi-contextual segregation refers to “the uneven spatiotemporal distribution of individuals from different social groups in various daily life contexts” [79]. The multiple contexts include various spatial contexts of individuals’ everyday lives (e.g., home, workplace and recreational places) as well as temporal contexts. This comprehensive conceptualization would help address the limitations of the traditional understanding of segregation as well as of many disparity issues that result from it, such as environmental injustice. It also calls for the development of new, fine-scale spatiotemporal and people-based methods that can incorporate such new concepts in order to untangle the dynamics and complexities of people’s segregation experiences.

### 5.2. A Notion of Multi-Contextual Segregation in Environmental Justice Research

Some studies have found only weak associations between the social and physical characteristics of people’s residential neighborhoods and those of their jobs, schools, shops, churches, recreational venues and other socially significant places [109,119,122,145]. This means that even individuals who live in the same residential neighborhood or are from the same household can experience different levels (or kinds) of social and environmental influences over the course of a day if they conduct daily activities outside of their residential areas. We argue that the notion of multi-contextual segregation is theoretically more meaningful and sounder than residential segregation for examining such differences.

Disparities in exposure to environmental stressors among different social groups can be intensified or mitigated depending on how greatly people are segregated at work or social/recreational venues and how much the environmental characteristics of these places differ from those of their residential neighborhoods. For example, if different social groups are more integrated in their workplaces or leisure-activity places than in their residential neighborhoods, then they may be equally exposed to similar levels of air quality in these non-residential places. If environmental quality of these non-residential areas is better than that of socioeconomically marginalized groups’ residential areas, being mobile may enable them to mitigate some of the environmental disadvantages of their residential neighborhood. This can ultimately reduce the disparity in total exposure to environmental health hazards among social and ethnic groups. Inagami et al. [146] found that the self-rated health of people in poor neighborhoods improved when they performed daily routines in non-residential areas. For socioeconomically marginalized people or racial minorities, however, long-distance commuting is often undertaken not by preference or choice, but rather by less-localized job markets and poor spatial access to job opportunities in every sector of the economy [147].

On the other hand, the disparity may increase when a particular group has limited access to workplaces or other activity places with better air quality than their residential neighborhoods, while other groups enjoy greater access. In general, whites or affluent people tend to have the social privilege and financial capacity (e.g., car ownership) to select their activity places [107]. They tend to voluntarily isolate themselves by limiting their mobility only to environmentally advantaged neighborhoods and avoiding disadvantaged urban spaces [105,148].

The same can be said for unequal exposure to beneficial environmental factors, such as exposure to urban green space, blue space, biodiversity, good aesthetics and community resources. Environmental injustice among social groups has been observed not only in exposure to environmental health hazards but also in exposure to environmental health benefits. Some studies have reported that minority groups or people from socioeconomically deprived communities tend to have poor access to and poor quality of green space, which may affect their health adversely [149,150]. These residence-based studies can be improved by considering human mobility and multi-contextual segregation. Shareck et al. [151] found that when people prioritize the environmental quality of places in which they conduct daily activities, their movements have a protective effect on their health. However, exposures to different beneficial and harmful sources tend to co-occur and are intertwined, having synergetic or hindering effects on health. Therefore, to better understand the effect of environment on health and health disparities, it is useful for future research to consider a wide variety of environmental exposure factors simultaneously and examine how the spatial and temporal contexts in which people are exposed to multiple environmental factors are segregated.

**Figure 2:**
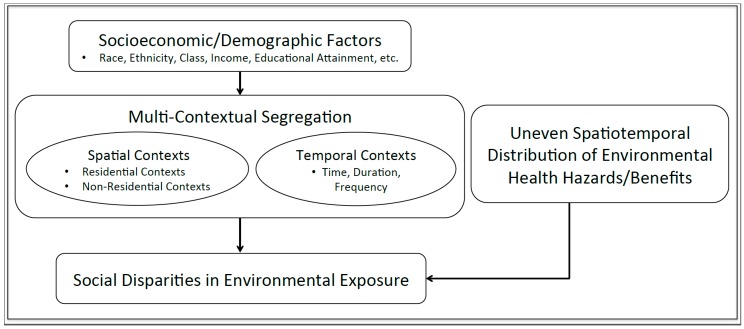
Conceptual framework for incorporating multi-contextual segregation in environmental justice research.

Figure 2 shows how multi-contextual segregation can affect social disparities in environmental exposure. This conceptual framework demonstrates that individuals’ spatiotemporal contexts can be shaped and segregated by social, cultural, economic and political processes based on socioeconomic and racial identities [105,110], causing multi-contextual segregation. Further, social disparity in environmental exposure arises when different social groups are segregated into various daily-life contexts as well as when environmental factors, such as air pollutants, are also spatially and/or temporally unevenly distributed (Figure 2). In conclusion, this paper argues that examining environmental justice issues through the lens of multi-contextual segregation could provide fruitful insights into the mechanisms underlying such disparity and strengthen the conceptual and analytical framework of environmental justice research.

## 6. Conclusions

This review paper suggests a future direction of quantitative environmental justice research. We argue that future research that seeks to link segregation and social disparities in environmental exposure should go beyond residence-based approaches and assess both segregation and environmental exposure at a fine spatiotemporal scale to address several methodological problems in residence-based methods. Integrating the notion of multi-contextual segregation into environmental justice studies, instead of the concept of residential segregation, would help examine the full range of segregated contexts in which people are disproportionately exposed to environmental health hazards or benefits. Such fine-grained research would provide more nuanced and fruitful insights into the complex socio-spatial mechanisms behind the perpetuating disparities in environmental exposure and health.

Kwan [69] argued that including time and human mobility into research design and analysis as critical dimensions would significantly enhance our knowledge about how people dynamically experience segregation and exposure to environmental health hazards/benefits over space and time. Time geography [152], which considers individuals’ mobility in space and time, can provide a useful conceptual and analytical framework for integrating environmental justice, multi-contextual segregation and Big Data-based environmental exposure/health research. The successful integration of a comprehensive and dynamic concept of segregation, fine-scale spatiotemporal data, fine-grained geospatial methods and advanced GIS technologies can strengthen existing segregation and environmental justice theories or highlight their limitations, while at the same time guide us toward developing new perspectives, critical insights, questions or theories that will enhance our understanding of various social issues. This would in turn help to address the broader social justice agenda at the intersection of urban segregation, environmental justice and health disparities and to develop more effective policies for contributing to desired societal changes.

## Figures and Tables

**Table 1:** The advantages and disadvantages of traditional residence-based approaches and fine-scale mobility-based approaches in segregation and environmental justice research.

		Checkerboard Problem	MAUP ^†^	UGCoP ^‡^	Advantages	Disadvantages
**Traditional Residence-Based Approaches**	**Aspatial measures of residential segregation**	O *	O	O	1. Easy to calculate and interpret. 2. Easy to obtain the required data (e.g., census population data). 3. Large sample size.	1. Incapability to capture segregation and environmental exposure that people experience in non-residential contexts. 2. One or more methodological problems, including the UGCoP. 3. Uncertainties in research results.
	**Spatial measures of residential segregation**	X **	X	O		
	**Residence-based assessments of environmental exposure**	N/A	O or X	O		
**Fine-Scale Mobility-Based Approaches**	**Spatiotemporal measures of segregation in various daily life spaces**	X	X	X	1. Assess people’s spatiotemporally varying segregation experience and environmental exposure. 2. Address or mitigate the three methodological problems. 3. Produce more reliable and robust analysis results.	1. Difficulty in obtaining high-resolution data due to high-cost and time-consuming collection process and privacy/data confidentiality issues. 2. Computational complexity of calculation. 3. Research participants may not be representative of all population groups, without thorough sampling plans.
	**Human mobility-based assessments of environmental exposure**	N/A	X	X		

**^†^** MAUP: modifiable areal unit problem; **^‡^** UGCoP: uncertain geographic context problem; * O: the problem likely exists in the measure; X **: the problem does not exist in the measure or is mitigated.
